# Risk and Safety in Radiographic Utilization: Evaluating the Evidence From Current Research

**DOI:** 10.1155/rrp/8843173

**Published:** 2025-11-29

**Authors:** Philip A. Arnone, Lauren M. Richards, Kalan C. Stittleburg, Michael T. Weiner, Alyssa K. Dennis

**Affiliations:** ^1^The Balanced Body Center, Matthews, North Carolina, USA; ^2^DeYoung Chiropractic, Wayland, Michigan, USA; ^3^Trillium Spinal Care, Altoona, Wisconsin, USA; ^4^Optimal Spine and Body, Alpharetta, Georgia, USA; ^5^Strong Spine and Body, Atlanta, Georgia, USA

**Keywords:** hormesis, linear no-threshold model, radiographic risk, radiographic safety

## Abstract

The 19^th^-century discovery of radiographic imaging techniques revolutionized healthcare, allowing doctors to indirectly “see” inside their living patients. It was not until the mid-20^th^ century that mutagenic effects from radiation exposure became a topic of concern. Since then, healthcare providers, scientists, and regulatory agencies have battled the question, “Does radiation exposure from radiographic imaging increase the risk of developing cancers and heritable conditions?” With no established answer, physicians must rely on the available evidence when assessing the risk versus benefit of having their patients x-rayed. This review aims to gather and consolidate the available evidence so physicians can make informed decisions when ordering tests that use x-rays. Popular topics such as the linear-no-threshold (LNT) model, radiation hormesis, and the as-low-as-reasonably-achievable (ALARA) principle are covered. Epidemiology studies, x-ray physics, and related basic sciences are reviewed.

## 1. Section A: Introduction

Radiographic procedures have had a long history of acceptance within healthcare as a valuable diagnostic tool; however, there has been debate in the literature throughout this time regarding the safety of these procedures. Physicians routinely perform radiographs as part of their typical scope of practice. The rationale for radiographic utilization varies, and some of these uses are for the purpose of identifying osseous abnormalities [1], evaluation after traumatic injury [2], and biomechanical analysis of the spine [3]. Research also demonstrates that radiographs are commonly utilized in cases of nonspecific low back pain, which may also include the purpose of patient reassurance [4]. There are publications that suggest the use of clinical radiography presents a danger to patient health and an increased risk of future disease [5]. More recent safety concerns are present in the research regarding the use of chest computerized tomography scans in the case of respiratory disease during the COVID-19 epidemic [6]. Conflicting research suggests that the concerns regarding radiographic procedures are outdated and nonscientific and suggests these procedures to be safe for clinical utilization [7]. There is conflict between the literature that suggests radiographic utilization presents increased risk and the literature that indicates there is limited concern; moreover, additional research is available that demonstrates there may be some associated health benefit from repeated exposure to low-dose radiation [8]. The aim of this study is to present a summary of the basic principles of radiographic exposure and to outline the various studies that support the conflicting theories fueling this debate. The goal is to provide a platform for practitioners to establish a clinical opinion regarding radiographic utilization in practice. These models and concepts are reviewed to provide the opportunity for physicians to use their clinical opinion to determine the best approach to radiographic utilization in practice. Understanding how radiation could affect patient health requires understanding research concepts such as the linear-no-threshold (LNT) theory, hormesis, the as-low-as-reasonably-achievable (ALARA) principle, and millisieverts and milligrays, which are summarized in this review. Publications are summarized supporting claims of physical and mental health risks associated with radiographic utilization and those suggesting that there is limited and/or no risk involved with periodic radiographic exposure.

### 1.1. Definitions

The following terms are pertinent to our discussion; therefore, they are defined below in Table 1.

On multiple occasions, our study uses measurement terms such as “high-dose” and “low-dose” when referring to radiation exposure. There is no established threshold for when a certain amount of absorbed radiation negatively impacts health; therefore, it is not possible to definitively define what constitutes a “high-dose” and what constitutes a “low-dose.” When our study uses these terms, we are quoting and citing verbiage from existing studies. Keep in mind that each study and regulatory agency has its own definitions and thresholds of “high-dose” and “low-dose.”

Though this study takes a neutral and scientific approach, the authors are based in and most familiar with the regulations and practices of the United States. Each physician must abide by their local regulations and their scope of practice for the use of ionizing radiation in diagnostic imaging.

## 2. Methods and Materials

Topics related to radiographic safety were reviewed in the literature utilizing studies from PubMed, the Index to Chiropractic Literature, and the Chiropractic Biophysics databases. Studies were selected that best and equally represented the various opinions regarding radiographic safety and risk associated with radiographic exposure in practice, including those relating to the literature supporting the LNT model, the literature contesting the LNT model, mental health, radiography procedures, hormesis, daily radiographic dosage, exposure, and calculated dose. Studies that were redundant and unrelated to safety were excluded. The authors chose not to set a specific date range when searching for relevant articles, as many of the studies relating to risk are over 20 years old, including the development of the LNT model. A total of 91 articles were reviewed, and 49 studies were utilized for this study.

### 2.1. Section B: Calculating Radiation in Daily Life

It is important to understand that there are many forms of “background radiation” exposure in the course of an average day and year. Background radiation is the natural exposure that humans receive incidentally from environmental sources such as radioactive elements in the ground, like radon, and from cosmic rays produced by the sun and outside the solar system [13]. During radiation exposure, one important factor is the amount of radiation absorbed, measured in milligray; moreover, millisievert is used to measure equivalent and effective doses, which are obtained by adjusting the absorbed dose expressed in milligray by the radiation weighting factor and tissue weighting factor, respectively. The type of radiation that the patient is exposed to and the type of tissues that are irradiated are also important factors. This is measured in milligrays (mGy). The millisievert (mSv), which is more commonly defined as the absorbed dosage, is an amount of radiation that takes the damaging properties of different types of radiation into account. The type of radiation with the potential for ill health effects is “ionizing radiation.” Ionizing radiation has enough energy to remove electrons from atoms, creating ions [13]. Forms of ionizing radiation include gamma rays, x-rays, and energetic (UVC or shortwave) ultraviolet light. The sources of these include natural elements in the earth, like radon, cosmic rays from the sun, and diagnostic medical imaging equipment, to name a few. Our review will focus on x-ray radiation, which is a form of ionizing radiation. Within the literature, there appears to be a large regional discrepancy between the amount of natural background radiation, which can range from 3 mSv annually in the U.S. to 260 mSv per year in other parts of the world. Despite the constant exposure to different daily levels of low-dose natural background radiation, there does not appear to be any evidence of associated health effects in the regions with exponentially higher levels of exposure [14]. Some examples of how dose varies depending on location and geology demonstrate that in northern Iran the yearly dose is 260 mSv/year and in Nigeria it is 90 mSv/year [15].

Practitioners can use this dosage calculator to educate patients on radiographic exposure in comparison to the exposure of radiation in daily activities, as these levels vary greatly depending on geographic location, what procedures one has experienced, the environment in which one works, and the number of procedures one has had [16].

Additionally, the Biological Effects of Ionizing Radiation (BEIR) VII report acknowledges that there is no known difference between natural and synthetic radiation. The latest report asserts that there are no known characteristics that can be identified to help distinguish between radiation from radon or radiation from a lumbar x-ray, for example [17].

This is perhaps best understood by identifying the mSv in radiographic procedures compared to radiographic exposure in daily life. As stated above, radiation exposure can occur in many forms, including cosmic radiation, ground radiation, airline flights, food, and water. Our bodies even produce their own small amounts of radiation [18, 19]. Some examples of daily dosage are defined in Tables 1 and 2.

### 2.2. Section C: Understanding the LNT Model

The literature that debates the risk versus safety of radiographs revolves around those who support and those who disagree with the LNT model. As a result, any physician who wishes to establish their clinical opinion on radiographic safety should consider understanding the LNT model and its history. The LNT model was first introduced in the 1950s, and it continues to be a focus of debate among the scientific community. Most researchers in the discipline do agree that high doses of ionizing radiation are harmful to the human body [20, 21]. However, the point at which something is defined as a high or low dose cannot be defined in literature (see Tables 3 and 4).

In 2006, the National Research Council of the National Academies of Sciences performed its seventh report on the BEIR VII Phase Two study. This report took over 4 years to complete and was undertaken by many nationally and internationally esteemed scientists, researchers, regulators, and professors. The project was funded by the Environmental Protection Agency, the Nuclear Regulatory Commission, and the U.S. Department of Commerce. This comes 15 years after BEIR V was completed. The goal of this most recent report was to update the old one on the health effects of low levels of low Linear Energy Transfer (low-LET) radiation, which is a measure of how much energy radiation deposits in a material as it travels through it, per unit of distance [17].

John Boice is a former president of the National Council on Radiation Protection and Measurements (NCRP). The NCRP gives recommendations to lawmakers for radiation regulation in the United States. His recommendations for radiation protection require an assumption of danger and harm, but he acknowledges there is no evidence that this danger or harm actually exists. He also acknowledges that the probability or risk of harm is very low, but he feels that it still exists [22].

One major point of contention with the LNT model is the extrapolation of high-dose data being applied to low-dose theory. In 2016, Sacks et al. published an article in the Journal of Biological Theory that called into question the legitimacy of the LNT model from a biological perspective. The authors point out that the LNT model lacks evidence, and it assumes that there is increased risk of cancer in the low-dose range. Additionally, it hinges on the idea of a cumulative effect from low-dose radiation that has a harmful impact on our body. They say the LNT model ignores our body's natural biological processes and that there is no evidence, epidemiological or otherwise, to support this claim of increased cancer risk [14]. In fact, low-dose extrapolation is biologically debated, particularly due to DNA repair after low-level ionizing radiation exposure and to hormesis, which is the stimulation of a biological system by low doses of a potentially harmful agent that could elicit positive adaptive effects.

In 1974, the U.S. Nuclear Regulatory Commission established the ALARA principle with the intention of protecting nuclear workers. Here is a quote from the USNRC website talking about the ALARA principle:

As defined in Title 10, Section 20.1003, of the *Code of Federal Regulations* [23], ALARA is an acronym for “as low as (is) reasonably achievable,” which means making every reasonable effort to maintain exposures to ionizing radiation as far below the dose limits as practical, consistent with the purpose for which the licensed activity is undertaken, taking into account the state of technology, the economics of improvements in relation to state of technology, the economics of improvements in relation to benefits to the public health and safety, and other societal and socioeconomic considerations, and in relation to utilization of nuclear energy and licensed materials in the public interest [24].

Although the limits vary depending on the body part affected, the USNRC has determined the annual total effective dose equivalent (TEDE) for the whole body is 5000 mrem [5] (5 rem) [25].

### 2.3. Section D: Research Supporting the LNT Model

Despite existing evidence that shows that low-dose radiation is not harmful to humans and that there exists a mechanism in the body that protects us from the potential damage of radiation, there are other studies that suggest that exposure to radiation can create higher long-term risk. There is existing evidence to suggest that overexposure can create a higher risk for various long-term health conditions when evaluating patient safety. Scoliosis patients are often studied for radiographic utilization, dosage, and long-term follow-up to assess for any risk of x-ray radiation issues, such as cancer, and it is found to be the gold standard for diagnosis and monitoring of adolescent idiopathic scoliosis [26]. Upon utilization of radiographs for scoliosis patients using a Milwaukee brace-treatment program, there was an increase in organ carcinogenic risk that ranged from 3.4 to fifteen per million (1.3% to 7.5%), except for breast tissue, which increased from 140 to 290 per million (110%) [27]. These patients received an average of 22 anteroposterior and lateral radiographs of the thoracic and lumbar spine over a 3-year brace program. They received these radiographs every 3–4 months. Posterior–anterior x-ray views reduced the risk of cancer by 3.8% and therefore support the claim that less radiation exposure for adolescent scoliosis patients will decrease cancer risks [27].

When studying the risks of cancer from radiographs of pediatric orthopedic patients, it was found that the risks for developing leukemia, breast cancer, or a heritable defect were 0.8%, 2.1%, and 3.0%, respectively, in the order of conditions previously stated, higher than any other baseline risks assessed [28]. These patients had radiographs due to idiopathic scoliosis, hip dysplasia, or leg-length discrepancy from 1980 to 1993 at Shriners Hospital in Spokane, WA. The author found that the risk of any cancer or hereditary defects was minimal and noted that the multiple x-rays taken in this study appeared relatively safe.

Using data from 1983–1990 with a 25-year follow-up, 170 patients with adolescent idiopathic scoliosis that received 16 radiographs during the treatment period had 9 patients develop cancer [5]. The highest rate was breast cancer and endometrial cancer, with this group having a relative risk of 4.8 for developing cancer compared to the normal age-matched Danish population. The limitations of this study did not have any information on genetic predisposition to cancers, previous smoking history, and other confounders associated with the development of breast and endometrial cancers.

A meta-analysis of 9 eligible studies that include 35,641 total participants between 1912 and 1990 found that scoliosis patients with 20 years of radiation exposure had elevated rates of cancer, breast cancer, and cancer mortality compared to the matched general population [29]. Since the data were collected from patients who underwent diagnostic imaging with older equipment, they received higher doses of radiation. The recent advancement of radiographic equipment has enabled the use of less radiation without compromising diagnostic image quality, as seen in digital radiography (DR) imaging [30]. In fact, low-dose imaging systems reduce radiation exposure by up to 45 times while maintaining image quality [31].

Another form of radiation used for diagnosis and management is computed tomography (CT). In a retrospective cohort study between the years of 1985 and 2002, patients less than 22 years of age were gathered to gather data for cancer incidence, mortality, and loss with the use of CT scans that did not have any previous cancer diagnosis [32]. The authors found 74 out of 178,604 patients were diagnosed with leukemia and 135 of 176,587 patients were diagnosed with brain tumors in their follow-up examination after receiving CT scans [32]. The authors found that cumulative doses of about 50 mGy might triple the risk of leukemia, and doses of 60 mGy might triple the risk of brain cancer. These numbers would equate to two to three head CTs with patients ranging from 1 to over 5 CT scans over a time period. These patients were monitored to see if there are any risks of cancer associated with the CTs used and if there is a threshold amount of radiation. The authors note that other methods of diagnosis and management that involve either no radiation or the lowest amount of radiation possible for a pediatric population should be considered to decrease any incidence rates of cancer that could develop due to ionizing radiation.

Compiling data from 1999 to 2003 with pediatric patients that used CT scans in Israel, it was found that there were 9.5 lifetime deaths out of 17,686 pediatric patients when followed throughout their life [33]. The authors noted that the use of pediatric CT scans may result in a small increased lifetime risk for cancer mortality and that the data are uncertain for any risks associated with low-dose radiation. Chest x-ray fluoroscopy is a method used for diagnosis and management of tuberculosis. Of 2573 women examined by x-ray fluoroscopy an average of 88 times during lung collapse therapy and followed for 30 years, 147 breast cancers occurred, with no excess breast cancer seen among 2367 women treated by other means [34]. The mean radiation dose to the breast was estimated to be 79 cGy (790 mSv) with strong evidence for a linear relationship between dose and breast cancer risk, with a relative risk at 1 Gy to be 1.61 times more likely to develop breast cancer. Due to the age of the article (1925–1954), it may not fully translate to the amount of radiation used today. Also, there are uncertainties associated with the patient orientation and length of time during fluoroscopy, making the conclusions harder to draw due to the variable amount of dosage the patients received.

Looking at data from Japanese atomic bomb survivors, the United Kingdom, and 13 other developing countries, there has been an increase of cases of cancer reported with x-ray use [35]. The results from this study show that in the United Kingdom, 0.6% of the cumulative risk of cancer to age 75 years could be attributable to diagnostic x-rays. In 13 developing countries, the attributable risk ranges from 0.6% to 1.8%. In Japan, it was found to be more than 3%. The methods used risk models, mainly from the Japanese atomic bomb survivors. This can confound the conclusion, as it may not be appropriate to compare the high-dosage atomic bomb survivors to patients undergoing x-rays.

A systematic review and meta-analysis was conducted of epidemiological studies from 2000–2019 that looked at patients younger than 22 years of age exposed to x-rays [36]. They found that prenatal exposure to either x-rays or CT scans led to no significant increased risk for cancers, leukemia, and brain tumors, whereas postnatal exposure to CTs led to an increased risk. X-ray exposure was not associated with increased risks of all cancers, lymphohematopoietic malignancies, or brain tumors. There is an association between exposure to low-dose radiation and an additional increase in breast cancer risk among high-risk women with a 1.3 times increased breast cancer risk—and higher risks for women exposed before the age of 20 [37]. These women used mammography or chest x-rays. When looking at a wider demographic than only women with breast cancer, the dose–response curve of low radiation to morbidity and mortality is unclear [38]. When looking at cancer risks with x-ray and nuclear medicine imaging, risk estimates should be better done through the individual organ dose assessments due to both genetic and environmental (including lifestyle) factors that can influence the cancer risk independent of radiation dose [39]. When looking at specific organs utilizing the BEIR VII proposed models, the thyroid had the highest cancer incidence in skull and cervical spine radiographic examinations [40]. In the chest and thoracic spine radiography, the risk of developing lung and breast cancers was notable. In the lumbar spine, pelvic, and abdominal x-ray examinations, the colon and bladder had the highest risk of cancer incidence. As discussed above, CT equipment technology has also advanced to reduce patient absorbed dose. The dental and chiropractic professions have adopted cone-beam computed tomography (CBCT) to visualize the anatomical regions of the head and neck. A typical AP cervical spine radiograph would cause a 0.12 mSv effective dose to the patient, while the same procedure from CBCT is 0.02 mSv [41, 42].

Multiple factors go into the safety of x-ray use, which include the amount of radiation, the equivalent dose, the tissues exposed to the x-rays, the age of the patient, and a lifetime dose of around 60 mGy, indicating a risk factor for cancer. The literature noted above shows there are incidences of cancer with radiation doses that support the LNT model.

### 2.4. Section E: Research Opposing the LNT Model

#### 2.4.1. The LNT Model: Hypothetical or Evidence-Based?

Many individuals and groups opposing the LNT model point to the lack of empirical evidence showing an actual increase in carcinogenesis in relation to low-dose radiation. The American Association of Physicists in Medicine (AAPM) is one such group. They feel that there is a lack of epidemiological evidence to reliably suggest an increase of cancer occurrence or mortality rates on radiation below 100 mSv annually. In addition, most diagnostic imaging is substantially below this 100 mSv threshold, so they feel that when it is appropriate, there is a potential for more harm by avoiding imaging because of unfounded concerns from radiation exposure [43].

A 2016 article published in the Journal of Technology in Cancer Research & Treatment by Siegel and Welsh further illustrates the projections of the LNT model in regard to radiation carcinogenesis. The authors concede that high doses and dose rates of radiation will increase cancer incidence, but it is irresponsible to extrapolate this information down for a theoretical increase in cancer incidence at low doses and dose rates of radiation in the 100–200 mSv exposure territory. They emphasize that the carcinogenic effects of low-dose radiation are completely theoretical [44].

The groups who stand behind the LNT model acknowledge this lack of evidence. The U.S. Department of Energy Low-Dose Radiation Research Program helped to publish an article in 2003 that acknowledges that there is no evidence to support the LNT model below 100 mGy [45]. John Boice from the NCRP agrees that epidemiological science is not capable of observing the accuracy of the LNT model with low-dose radiation [22]. The BEIR VII committee is in agreement that science cannot provide evidence of an increased cancer risk in humans at low-dose rate radiation [17].

#### 2.4.2. Can the LNT Model Be Proven Correct or Incorrect?

One of the more consistent arguments in support of the LNT model is that it has been the established theory since 1959. The United States Environmental Protection Agency (USEPA) feels that there is a lack of evidence to overturn the LNT model from a biological mechanism perspective and should therefore be assumed to be accurate [20, 46]. The BEIR VII report has the perspective of the LNT model being a given and that it is a convenient point to start the radiation protection discussion [17]. John Boice agrees that the LNT model cannot be scientifically verified in the low dose range and that it is purely an assumption but that there are no better alternative theories regarding radiation carcinogenesis based on our present understanding [22].

In contrast to the authoritative perspectives that suggest the LNT model is appropriate based on current knowledge, a 2018 publication in the Dose Response Journal suggested that the lack of evidence for the LNT should be cause to reject this model. Rather than having to prove a relationship does not exist, it should be the responsibility of the LNT model to prove itself correct prior to its acceptance. They contend that science is conducted by assuming a null hypothesis, meaning no relationship, and then those who suggest a relationship exists must show evidence of that relationship. The authors feel that assuming a relationship exists between any amount of radiation and an increase in cancer incidence, like the LNT model does, is unscientific because it cannot be proven false [20]. One comparison that has been utilized to describe the flaws in the cumulative theory of LNT is considering a chef who frequently cuts his fingers, causing a slight loss of blood, is at high risk from this slight loss because if this happened enough times, the cumulative loss of blood would be enough to cause death [14].

Radiation, Science, and Health, Inc. offers $1000 for one report in English with scientifically acceptable evidence of harm (increased cancer death rate or decreased average lifespan) from low-dose irradiation in normal (not immune deficient) humans or laboratory animals [47].

#### 2.4.3. Other Plausible Theories Related to Low-Level Radiation Impact

In 2005, an expert advisory board from the French National Academies of Science and Medicine rejected the LNT model and instead adopted the hormesis model for radiation [48]. TD Luckey describes what hormesis is in his 2006 article entitled *Hormesis: The Good, the Bad, and the Ugly*, as a stimulation of a bodily system that uses low doses of an agent. He suggests that different-sized doses may result in different system responses; a large dose and a small dose may cause exactly opposite results. A specific amount of the dose will separate these results going opposite directions, and that dose is called the “zero equivalent point” (ZEP). This dose amount could also be called a threshold. According to Luckey, any dose below the ZEP, or threshold, can be considered a low dose [47].

In the mid-1990s, 15 research projects were performed at 10 Japanese universities and concluded that low-dose radiation stimulates many body processes and leads to improved health outcomes [47]. According to T.D. Luckey, there are over 3000 articles in the scientific literature that show low-dose radiation can have potential biological benefits to living cells. [49].

In a retrospective study regarding 8600 cleanup workers at Chernobyl, the researchers found a 12% lower rate of cancer than the general Russian population despite an average exposure of 5 cGy (50 mSv); however, potential confounding variables could not be well assessed, such as different genetic, lifestyle, and environmental risk factors [47]. In Taiwan, a study was published in 2004 on approximately 10,000 residents who were unknowingly exposed to additional radiation in their apartment buildings for 9–20 years. These residents were studied and found to have a reduced cancer incidence rate by more than 95% [50, 51]. The researchers concluded that these individuals with low levels of long-term radiation exposure saw a much lower mortality rate from cancer when exposed to 50 mSv of radiation per year [50, 51].

The hormesis model works together with the “bystander effect,” which takes place when a cell is nearly missed by radiation but near another cell that is radiated; this bystander cell can either add to biological damage or suppress it. The protective effect is found to take place in the dose range of 0.1–100 mGy of low-LET radiation [51].

### 2.5. Section F: Radiographs and Mental Health

In reviewing current evidence about the use of radiographic utilization in chiropractic practice, it is been observed that one common reason for referrals is to reassure patients that there is no underlying pathology. Patients often expect imaging when managing back pain because they believe it helps diagnose the source of their discomfort and guide treatment decisions, and as a result, many physicians order radiographs to satisfy patient expectations [52]. Research has demonstrated that this routine imaging may actually be linked to a lower sense of well-being and overall diminished health status [40]. In some cases, inconclusive x-ray findings or suspicions of pathology can lead to unnecessary follow-up investigations, heightening anxiety for the patient until a diagnosis is confirmed or ruled out. Studies, such as one by Wnuk et al., found that a significant percentage of imaging results indicating possible cancer or infection turned out to be false positives. For patients without any serious indicators, additional information from an x-ray offers minimal health benefits and may, in fact, cause undue concern, leading to care that is ultimately of low value [53]. It is important to note that while studies were available in primary care practices, there were no such studies available in chiropractic or orthopedic practice.

While many health conditions have established risk algorithms, there is less available data on how to communicate effectively with patients about these risks, the benefits of treatments, potential adverse effects, and whether the way this information is conveyed could influence patient outcomes. One study examining how radiologists communicate radiographic results suggested that withholding information could increase the likelihood of litigation, although the psychological effects of such decisions were also acknowledged [54]. This research did not evaluate the methods of communication in detail, but it did highlight the need for further exploration of this issue. Another study discussed improving communication through structured reporting between radiologists and referring physicians but did not address how this affects patient health [55]. Additionally, the literature revealed a significant gap in understanding how physician reporting influences patients' health in the context of chiropractic or orthopedic practices. While some studies in primary care and radiology have explored these topics, no similar research was found in chiropractic or orthopedic settings. That said, there are studies in the cardiovascular field that suggest how different communication methods can influence patient outcomes. There are also limited studies on how radiographic reporting affects patient health; there is insight regarding the effect of how a physician delivers a report and patient health within the cardiology research [56]. When it comes to cardiovascular disease (CVD), one of the leading causes of death in the United States, a “four-part model for effective shared decision-making” was proposed by prestigious medical institutions such as Duke, Northwestern, and Johns Hopkins. The model stresses that physicians play a central role in determining what information is communicated and how it is delivered. The guideline recognizes that, although shared decision-making and patient engagement are vital to treatment, there is limited data on how these discussions should be conducted. In the context of CVD, it is crucial for clinicians to inform patients about their risk, but ultimately, the patient should be the one to make decisions. Ongoing discussions about prevention are necessary to ensure patient adherence, address changes in risk, and adjust to shifting patient preferences [56].

A qualitative study in Northern Portugal assessed the experiences of diabetes patients and their care providers. The findings pointed out various factors that could strengthen the patient–provider relationship, encourage patient involvement, and enhance communication skills [57]. The study also highlighted that patient-centered communication is linked to better disease knowledge, improved self-care, enhanced quality of life, and better control of metabolic measures. However, despite these recommendations, studies such as the Diabetes Attitudes Wishes and Needs (DAWN2) survey suggest that patient-centered care is often lacking, with many patients' psychosocial needs unmet due to communication breakdowns. These failures are one of the most common complaints among patients and contribute to harm [57]. The literature on communication offers valuable guidance on how to improve patient-centered care, focusing on aspects such as fostering healing relationships, decision-making, providing information, addressing emotions, and supporting self-management.

### 2.6. Section G. Discussion

The question that is hard to determine is at what point does radiology become dangerous? The conclusion of those who suggest radiographic utilization is dangerous would suggest if radiation is deemed dangerous in very high doses, then the only acceptable safe dose is zero. Given this conclusion, it seems the only way to ensure patient safety would be to avoid radiographic use altogether. Unfortunately, in many forms of health care there is some degree of associated risk. Radiographic procedures have been a part of healthcare for many decades, and there will continue to be the need for radiographic utilization in certain cases. Without an exact point at which dosage becomes dangerous, physicians must establish a clinical opinion based on the current literature regarding risk versus value.

A concern arising from those who support the LNT model is “cumulative dose.” The theory is that radiation exposure causes DNA alterations in cells, and repeated exposure will lead to mutations, which then turn into cancer. This seems reasonable, as evidence suggests that even small doses of radiation can lead to changes in DNA [58]. This argument ignores the natural physiology of every living organism, as supported by the hormesis models. Living organisms experience regular DNA changes but have an inherent ability to repair and heal them [20]. There are substantially more natural DNA alterations from normal bodily processes than there are alterations as a result of most instances of radiation. One study has suggested that there is a great degree of complexity regarding carcinogenesis due to the rarity of its occurrence [59]. There are flaws in these studies that support the LNT model. These studies date back to the 1900s; some only studied pediatric populations, some of whom already had cancer and were unable to have a proper health history to rule out other causes of cancer, and some studies use mathematical predictive models as estimates based on equations to predict cancer risks. This can lead to non-objective data and instead projecting risks. The literature did not explore hormesis and how the body integrates low-level radiation. Correlative data from the literature above cannot claim that radiographs will cause cancer and instead can only predict a proposed risk. Data do support the risk of x-ray radiation to support the LNT model. However, no studies have shown a cause-and-effect relationship, which can lead to the strength of the data being questioned.

As advancements in imaging equipment improve, a practitioner's decision to utilize imaging in practice may evolve. The equipment and imaging have demonstrated improvements in localization, precision, collimation, decrease in exposure time, and appropriate dose ratio. Practice guidelines today drawn from equipment performance in the 1960s may prove to be outdated [60]. According to a 2016 retrospective study comparing typical technical parameters from 1939 to 2010 and comparing 14 common radiographic procedures: Advancements in technology, imaging protocols, and protective measures have led to a significant reduction in estimated organ doses over time [60]. This study was performed by representatives from the National Cancer Institute, National Institute of Health, Food and Drug Administration, and Division of Cancer Epidemiology and Genetics. As a result of enhancements to radiographic equipment, modern radiographs demonstrate significantly lower levels of radiation in comparison to the equipment utilized in prior decades, including the equipment used in many studies that demonstrate radiographic risk [60].

Hormetic changes, or the beneficial use of radiation to stimulate cellular repair, provide insight into the physiological aspect of radiology. Epidemiological studies of this population indicate that low doses of radiation did not increase cancer risk and may, in fact, be associated with a protective effect [20]. USEPA goes on to comment, ionizing radiation causes complex DNA damage that is difficult to repair, leading to error-prone repair and mutations. This damage is unique to radiation, and no threshold for radiation-induced mutations has been observed, meaning even low doses can accumulate risk over time [20]. This statement relies on a biological plausibility argument to support the use of the LNT dose–response model in low-dose, low-dose-rate (LDDR) environments. However, a biologically plausible argument based on more recent scientific evidence suggests that extensive protective biological processes are initiated upon initial DNA damage to prevent potential development of cancer (e.g., cellular and tissue-level defense mechanisms including not only DNA damage repair but also apoptosis, premature terminal differentiation, and immunosurveillance [20]. The rate of DNA alterations caused by the body's natural oxidative metabolism far exceeds the rate of DNA changes resulting from background radiation or most other radiation exposures [44].

The USEPA's response implies that any DNA damage not repaired with perfect accuracy leads to an increased cancer risk. However, current evidence does not support this claim. Cellular DNA repair systems respond to both radiation-induced damage and the ongoing background damage caused by normal metabolic processes, such as oxygen metabolism. If the total DNA damage following radiation exposure is actually lower than the pre-existing level of spontaneous damage, it is biologically plausible—and consistent with existing science—that radiation may not increase cancer risk (supporting the idea of a threshold) and could even reduce it (supporting the concept of hormesis) [20].

As research is published, it allows the standard of care to support the most up-to-date information in terms of radiology use. Prior communication and techniques may prove to be flawed. When patients are told that radiology is harmful, this can lead to radiophobia. There are clinical reasons to take plain film radiology that benefit the patient and doctor. Highlighting that someone is overweight can be very damaging to one's mental health. Determining that someone has cancer, heart disease, type 2 diabetes, spinal or joint problems, and so on can all be mentally damaging. The problem that exists is the way in which the information is being communicated to the patient. In all forms of healthcare, how the patient responds to the patient's report is influenced by the physician's skill in giving that report. If delivering the report causes mental distress, the responsibility may lie with the physician's communication, not with the tests or the results, respectively. There are no audits done on what and how information is being discussed from doctor to patient, so it is challenging to draw conclusions on how taking x-rays truly affects mental health in a health-related profession. Further studies are required to determine the effect of physician reporting and patient response.

Risk remains a complicated concept with many variables that make it difficult to directly measure. In the studies that show a positive correlation between x-ray exposure and the risk of cancer, there is no causal evidence that supports this claim. Correlation can never prove causation, but instead, it can only show a relationship between the two. The relationship may not be causal in nature—therefore, all claims based on the studies noted above that say radiation doses will increase cancer risk cannot be fully substantiated. In addition, these studies used mathematical models to estimate the cancer risk. These estimations are not proven data but can be estimated using formulas to assess the risk that patients will get cancer due to radiographic exposure. The scientific understanding of the effects of radiation exposures has evolved since its discovery in the late 19th century. The scientific information supporting the use of the LNT model for LDDR radiation environments developed over the past 60 years but is mainly extrapolated from high-dose, high-dose-rate (HDDR) environments. There are many confounding variables when studying the incidence of cancer and its relationship to radiation exposure. The literature does not include other variables such as weight, smoking, previous history of cancer, and proper health history to rule out other causes of cancer. Due to multiple variables that can increase cancer risk, it makes it difficult to prove causation that radiation at a certain level will lead to cancer when other variables are considered.

The review of literature may change the course of diagnostic imaging and treatment in many healthcare facilities. While the simplicity of the LNT model for risk assessment has traditionally been viewed as reasonably conservative, its utilization may encourage the belief that the smallest amount of radiation poses an undue risk [20]. A few key factors to consider when evaluating the risk associated with radiographic procedures:

• Whenever something is dangerous in high doses, the only way to guarantee safety is to avoid the procedure.
• Of 2573 women examined by x-ray fluoroscopy an average of 88 times during lung collapse therapy and followed for 30 years, 147 breast cancers occurred, with no excess breast cancer seen among 2367 women treated by other means [34].
• A systematic review found that prenatal exposure to either x-rays or CT scans led to no significant increased risk for cancers, leukemia, and brain tumors, whereas postnatal exposure to CTs led to an increased risk [36].
• Epidemiological studies of this population show that low-dose radiation did not increase cancer risk and may even be linked to a protective effect [20].
• Humans are continuously exposed to low-dose-rate radiation from natural background sources every moment of the day. In the United States, the average annual exposure is about 3 mSv, with levels reaching up to 260 mSv per year in some regions of the world, depending on location. Despite these varying levels of natural or other low-dose radiation exposures, no adverse health effects have been documented in any population globally [14].
• Currently, epidemiological evidence does not conclusively show an increase in cancer incidence or mortality from radiation doses below 100 mSv. Since diagnostic imaging typically involves doses well below this level, medically appropriate imaging is expected to provide benefits that far outweigh any minimal potential risks. — American Association of Physicists in Medicine [42].
• In general, epidemiology lacks the statistical power to reliably detect or measure cancer risks from low-LET (linear energy transfer) radiation at doses below approximately 100 mGy [57].
• The LNT model is a theoretical assumption that has not been—and cannot be—scientifically confirmed for low-dose radiation exposure. — John Boice, president of the National Council on Radiation Protection and Measurements [21].
• More than 3000 scientific studies have demonstrated that low-dose radiation can have stimulating and/or beneficial effects across a broad range of organisms, including microbes, plants, invertebrates, and vertebrates. — T.D. Luckey [48].
• The experience of these 10,000 individuals suggests that long-term exposure to radiation at approximately 50 mSv per year significantly lowers cancer mortality, a leading cause of death in North America [20].
• A single mutation alone does not cause cancer. Over a lifetime, each gene in an individual is likely to experience around 10^10 mutations. The mutation rate caused by typical natural background radiation, approximately 3–30 changes per year, is estimated to be about 2.5 million times lower than the normal spontaneous mutation rate [14].

## 3. Limitations

There are significant limitations that exist with the topic of radiographic risk and safety. There are too many parameters that increase the risk of disease to be able to isolate the unique risk of low-dose exposure on future patient health. Our study lacks the depth of a comprehensive review and is intended to highlight the various opinions that surround radiographic risk and safety. Studies that discuss increased risks of cancer due to radiographs are mostly based on older studies and are difficult to repeat, as they require tracking patient history over long periods of time. As the mSv of modern radiographs are significantly lower than older procedures, it is difficult to compare the data. Regarding mental health, there is a lack of literature relating to the type of reporting and how varied styles of reporting can affect patient health.

## 4. Conclusion

While extreme exposure to radiation has been demonstrated to be harmful, the current literature does not provide definitive evidence that radiation exposure from diagnostic imaging increases the risk for disease. While in many cases, the exposure from diagnostic imaging is similar to that from daily exposure, there is also no definitive evidence to prove that medical imaging is completely safe. A threshold level of radiation exposure that leads to adverse health effects has not, and likely cannot be, determined. The process of hormesis exists to repair bodily damage from radiation exposure; however, the extent to which this mitigates risk from exposure is also inconclusive. Improved technology has significantly reduced the amount of exposure from medical imaging over the past decades, which has likely inherently reduced risk, but the effect of this can also not be determined. To date, radiation less than 100 mSv per year cannot be proven to increase risk, and due to the limitations of researching this topic, a definite conclusion on the risks of diagnostic imaging radiation exposure cannot be established in the current literature. Considering that disease risk is related to a combination of genetic factors and environmental influences, and that radiation exposure caused by radiographic examinations is only one of many interplaying variables, physicians must understand and assess the risks of the procedures that they utilize. Our study reviews the health risks of ionizing radiation exposure, which is used in diagnostic medical imaging, and finds that the current research does not provide clarity regarding a specific threshold when radiation becomes harmful. Developing a clinical opinion regarding the levels of risk associated with radiation exposure from diagnostic imaging requires an understanding of the data that indicates risk, the current dosage of exposure from diagnostic imaging, the comparative data on daily exposure, hormesis, and the concept of exposure threshold, as all are reviewed in this study.

## Figures and Tables

**Table 1:** Definitions.

Term	Definition
Linear-no-threshold (LNT) theory	A dose–response model is used in radiation protection to estimate harmful effects from ionizing radiation. It assumes that any amount of radiation exposure carries a risk of causing ill health effects and that the risk increases linearly with the dose [9]
Hormesis	A phenomenon in which low doses of a substance or stressor can have beneficial effects, while higher doses can be harmful [8]
As-low-as-reasonably-achievable (ALARA) principle	ALARA is a principle that emphasizes making every reasonable effort to keep radiation doses as low as possible without sacrificing diagnostic image quality [10]
Millisieverts	A unit of measurement for the equivalent dose and effective dose, which are obtained by adjusting the absorbed dose expressed in milligray by the radiation weighting factor and tissue weighting factor, respectively [11]
Milligrams	A unit of measurement for radiation dose absorbed by the human body [11]
Absorbed dose	The amount of energy deposited by radiation in a mass [12]
Equivalent dose	The amount of radiation absorbed by a specific organ. Equivalent dose is measured in millisieverts (mSv) [12]
Effective dose	The amount of radiation absorbed by the whole body. Effective dose is measured in millisieverts (mSv) [12]

**Table 2:** Relative doses from radiation sources.

Source	Millirems (mrem)	Millisieverts (MSv)
Whole-body CT	1000	10
Radon in the average U.S. home	228	2.28
Annual cosmic radiation living in Denver	80	0.8
Annual cosmic radiation living at sea level	30	0.3
Terrestrial radioactivity	21	0.21
Upper gastrointestinal x-ray	600	6
Head CT	42	0.42
Radiation in the body (annual)	29	0.29
Mammogram (single procedure)	42	0.42
Terrestrial radioactivity (annual)	21	0.21
Chest x-ray (single procedure)	10	0.1
Living near a nuclear power station (annual)	< 1	< 0.01

*Note:* Table 2 from U.S. EPA O. Radiation Sources and Doses [Internet]. 2015 [cited 2024 May 1]. Available from: https://www.epa.gov/radiation/radiation-sources-and-doses.

**Table 3:** Calculated radiation dose for the whole body from different exams in the pediatric population.

Source	Effective dose (mSv)
Plain film examination	
** **Chest (anteroposterior and lateral)	0.06
** **Skull (anteroposterior and lateral)	0.04
** **Pelvis (anteroposterior)	0.7
** **Lumbar spine (anteroposterior)	0.7
** **Mammogram (four views)	0.7
** **Abdomen	1.2
** **Barium swallow	1.5
** **Barium enema	7
CT examination	
** **Head	2
** **Chest	8
** **Abdomen	10
** **Pelvis	10
** **Angioplasty	7.5–57

All doses are based on one procedure or annual exposure.

*Note:* Table 3 from Jaffurs D, Denny A. Diagnostic Pediatric Computed Tomographic Scans of the Head: Actual Dosage versus Estimated Risk: Plastic and Reconstructive Surgery. 2009 Oct; 124 (4):1254–60.

**Table 4:** Summary of research.

Study	Dose	Population	Outcome
Risks of Exposure to x-rays in Patients Undergoing Long-Term Treatment for Scoliosis [26]	22 AP and lateral radiographs of the thoracic and lumbar spine over 3 years every 3–4 months.	13 teenage girls with idiopathic adolescent scoliosis.	Increase in organ carcinogenic risk that ranged from 3.4 to 15 per million. For breast tissue, the risk increased from 140 to 290 per million. There was a 3.8% reduction of risk with PA x-rays.
The Risk of Carcinogenesis from Radiographs to Pediatric Orthopedic Patients [27]	The exact dose was not reported.	Patients at Shriners Hospital in Spokane, WA, from 1980 to 1993. Children were treated for idiopathic scoliosis, hip dysplasia, or leg-length discrepancy.	Risks for developing leukemia, breast cancer, or a heritable defect were 0.8%, 2.1%, and 3.0%, respectively compared to baseline risks. Risks of cancer or hereditary defects were minimal, and x-rays appeared relatively safe.
Incidence of Cancer in Adolescent Idiopathic Scoliosis Patients Treated 25 Years Previously [5]	16 x-rays during treatment.	170 patients with adolescent idiopathic scoliosis from 1983 to 1990 with a 25-year follow-up.	9 patients developed cancer in the 25-year follow-up.
Cancer and Mortality Risks of Patients with Scoliosis from Radiation Exposure: A Systematic Review and Meta-Analysis [28]	Average full-spine x-ray was 23.13, with the estimated mean cumulated radiation dose of the breast being 11.35 cGy.	35,641 participants between 1912 and 1990. 18,873 patients with scoliosis and 16,768 controls were used.	Patients with 20 years of radiation exposure had elevated rates of cancer, breast cancer, and cancer mortality compared to the matched general population.
Radiation Exposure from CT Scans in Childhood and Subsequent Risk of Leukemia and Brain Tumors: A Retrospective Cohort Study [31]	Varying dosages depending on CT scans. See results for what the authors believe the threshold amount to be for cumulative doses.	Retrospective cohort study between 1985 and 2002 with patients less than 22 years of age (*n* = 178,604 when looking at leukemia risks and *n* = 176,587 when looking at brain tumor risks).	Cumulative doses around 50 mGy might triple the risk of leukemia, and 60 mGy might triple the risk of brain cancer.
Excess Lifetime Cancer Mortality Risk Attributable to Radiation Exposure from Computed Tomography Examinations in Children [32]	Dose (mGy) ranged from 24 to 130 depending on age and region (brain or stomach) of the body tested with CT.	Pediatric patients from 1999 to 2003 with 17,686 scans were calculated.	Pediatric CT scans in Israel may result in a small but not negligible increased lifetime risk for cancer mortality.
Frequent Chest X-ray Fluoroscopy and Breast Cancer Incidence Among Tuberculosis Patients in Massachusetts [33]	An average of 88 x-ray fluoroscopy exams was used, with the mean radiation dose estimated to be 790 mSv.	2573 women for 30 years	147 breast cancers occurred, with no excess breast cancer seen with the other 2367 women treated by other means.
Risk of Cancer from Diagnostic X-rays Estimates for the UK and 14 Other Countries [34]	Modeling study based on large-scale public health data. Therefore, dosages and population size were not used for this type of study.	N/A	0.6%–1.8% of the cumulative risk of cancer to 75 years is attributable to x-rays in the United Kingdom and 13 other developing countries. In Japan, the risk was more than 3%.
Early Life Ionizing Radiation Exposure and Cancer Risks: Systematic Review and Meta-Analysis [35]	No dosages were specifically used, as this was a systematic review and meta-analysis.	Systematic review and meta-analysis from 2000–2019 of patients younger than 22 years of age exposed to x-rays.	CT exposure in childhood appears to be associated with increased risk of cancer, while no significant association was observed with diagnostic radiographs.

## Data Availability

The datasets generated during and/or analyzed during the current study are not publicly available but are available from the corresponding author on reasonable request.

## Endnotes

^1^For reference, 5000 mrem equals 50 mSv [56].
